# In-Situ GISAXS Study of Supramolecular Nanofibers having Ultrafast Humidity Sensitivity

**DOI:** 10.1038/s41598-017-00309-2

**Published:** 2017-03-21

**Authors:** Arpan Bhattacharyya, Milan K. Sanyal, Umesha Mogera, Subi J. George, Mrinmay K. Mukhopadhyay, Santanu Maiti, Giridhar U. Kulkarni

**Affiliations:** 1 Saha Institute of Nuclear Physics, 1/AF Bidhannagar, Kolkata, 700 064 India; 2 Jawaharlal Nehru Centre for Advanced Scientific Research, Jakkur, Bangalore 560064 India; 3 Centre for Nano and Soft Matter Sciences, Jalahalli P.O., Bangalore, 560013 India

## Abstract

Self assembled nanofibers derived from donor-acceptor (D-A) pair of dodecyl methyl viologen (DMV) and potassium salt of coronene tetracarboxylate (CS) is an excellent material for the development of organic electronic devices particularly for ultrafast response to relative humidity (RH). Here we have presented the results of *in-situ* grazing incidence small angle x-ray scattering (GISAXS) measurements to understand aridity dependent self reorganization of the nanofibers. The instantaneous changes in the organization of the nanofibers was monitored with different equilibrium RH conditions. Additionally formation of nanofibers during drying was studied by GISAXS technique – the results show two distinct stages of structural arrangements, first the formation of a lamellar mesophase and then, the evolution of a distorted hexagonal lattice. The RH dependent GISAXS results revealed a high degree of swelling in the lattice of the micelles and reduction in the distortion of the hexagonal structure with increase in RH. In high RH condition, the nanofibers show elliptical distortion but could not break into lamellar phase as observed during formation through drying. This observed structural deformation gives insight into nanoscopic structural changes of the micelles with change in RH around it and in turn explains ultrafast sensitivity in its conductivity for RH variation.

## Introduction

**Figure 1:**
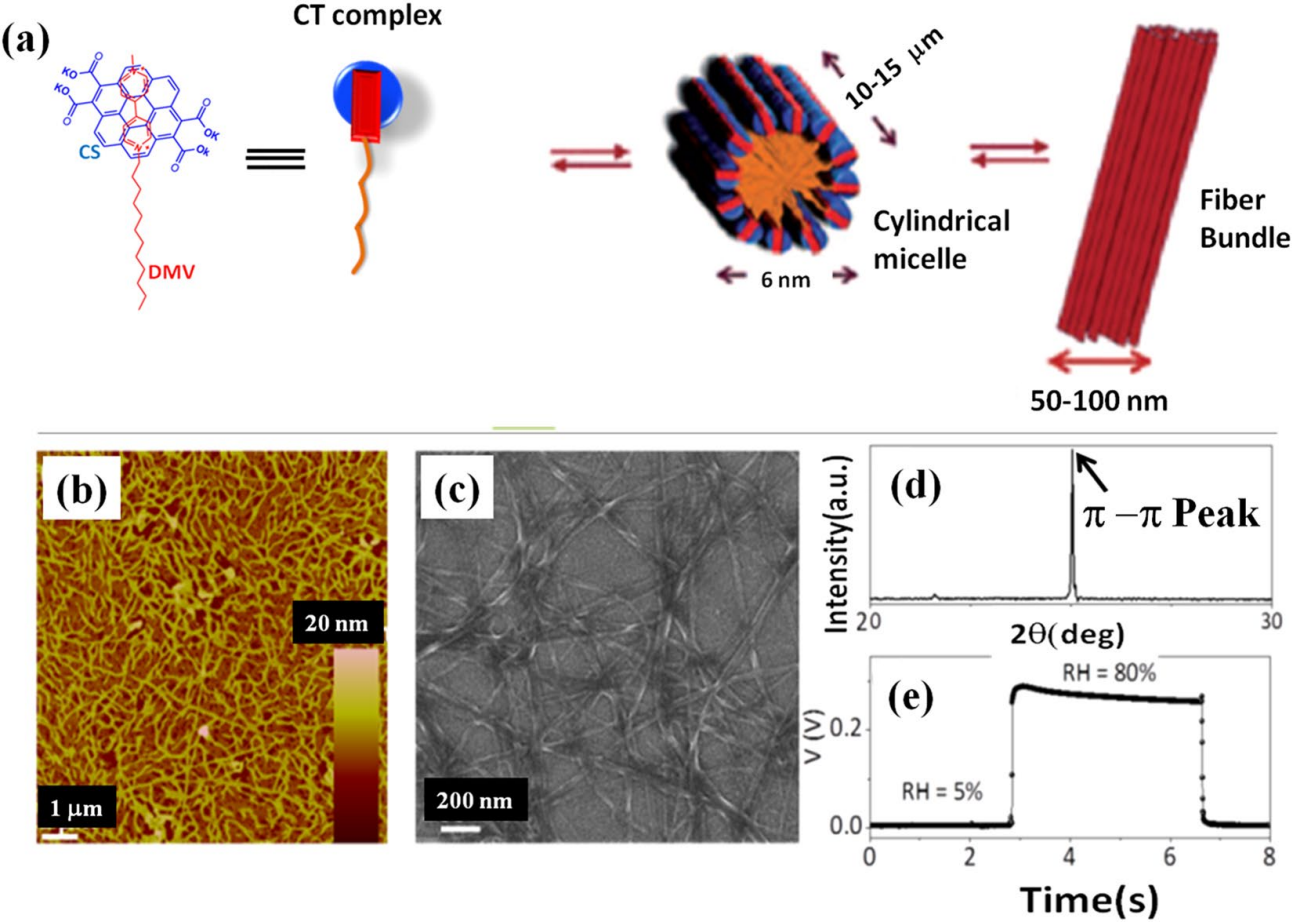
(**a**) Schematics of the self organization mechanism of DMV-CS nanofibers, initially single columnar micelle are formed and then these micelles aggregate further to form 50–150 nm diameter nanofibers. The molecular structure of DMV and CS molecules are also shown. (**b**) Tapping mode AFM image of DMV-CS nanofiber film dried after spin coating on a substrate. (**c**) FESEM image of nanofiber film after drying exhibit 1D nature of nanofibers. (**d**) X-ray diffraction data of the dried film measured at ambient conditions (26 °C, 40% RH). The peak at 2θ = 25.03° corresponds to π-π distance (3.555 Å) between DMV and CS charge transfer units. (**e**) RH dependent conductivity of DMV-CS nanofiber conditions showing the rapid change in its conductivity when RH was switched between 5% and 80%.

Self organization processes in supramolecular materials mimics the biological world in many ways as non-covalent intermolecular forces in both cases generate architectural complexity with few building blocks^1–3^. However, understanding^4–7^ of the self organization processes in presence of foreign species is a challenge but is crucial in developing new functional devices based on these self assembled materials. Development of organic materials using supramolecular design stratergies^8–10^ to achieve novel functionalities has been in the forefront of research interest^11–15^. Supramolecular materials are relatively new class of organic materials which are considered as promising candidates for various applications such as solar cells^16–19^, sensors^20, 21^, FETs^11, 12, 22–26^ etc. Interactions in these materials benefit from the intermediate size regime (few tens of nm to µm) that lies between single molecule (few nm) and polymer thin films (~µm)^11^ which enable easy fabrication. In this context, donor acceptor (D-A)^27–29^ molecules gain importance as the charge transfer interactions can be fine tuned by controlling the nature of organization using external influences^30, 31^ leading to different morphologies including 1D nanostructures^8, 22, 32^. Dodecyl methyl viologen (DMV) and potassium salt of coronene tetracarboxylate (CS) are one such class of D-A pairs which are designed to form cylindrical micellar structure in water as shown in Fig. 1. The face to face co-assembly process of DMV-CS molecules is solution mediated in which cylindrical micelle of amphiphilic bilayers of about 6 nm diameter^33, 34^ are formed with two individual charge transfer moieties arranged head to tail. Individual micelles further coalesce into higher order structures^35^ presumably due to their surface charges to form nanofibers with 50–150 nm diameter and also thicker fibers (refer Fig. 1(b) and (c)). This self assembly process is integrative in nature and occurs due to the interaction of the hydrophilic head and the hydrophobic tail with water as medium.

It has been shown that the relative humidity (RH) does have a major role in determining the properties of DMV-CS self-assembled nanofibers. The electronic properties of this nanofibers have been investigated earlier as active material in FET devices^36^ and high values of charge carrier mobility were obtained. It was also observed that the sensitivity of the nanofiber-conductivity is very fast to a small change in RH and it is possible to develop an ultrafast RH sensor with these fibers. Using UV-Vis, XRD and AFM measurements, it has been found that the π-π interaction length - the *d* spacing (refer Fig. 1(d)) corresponding to the π-π bond of DMV-CS moieties - shortens with increasing RH giving a significant change (refer Fig. 1(e)) in the conductivity^37^. Although ultrafast RH sensor based on similar supramolecular system was also reported^38^, detailed study of nanoscopic structural changes in the self-assembled organization of these molecules as a function of aridity has not been carried out till date. Here we report for the first time the aridity dependent change in the self-assembled internal structure of the DMV-CS nanofibers during formation through drying of dispersion and also by exposing these nanofibers to different equilibrium RH conditions.

**Figure 2:**
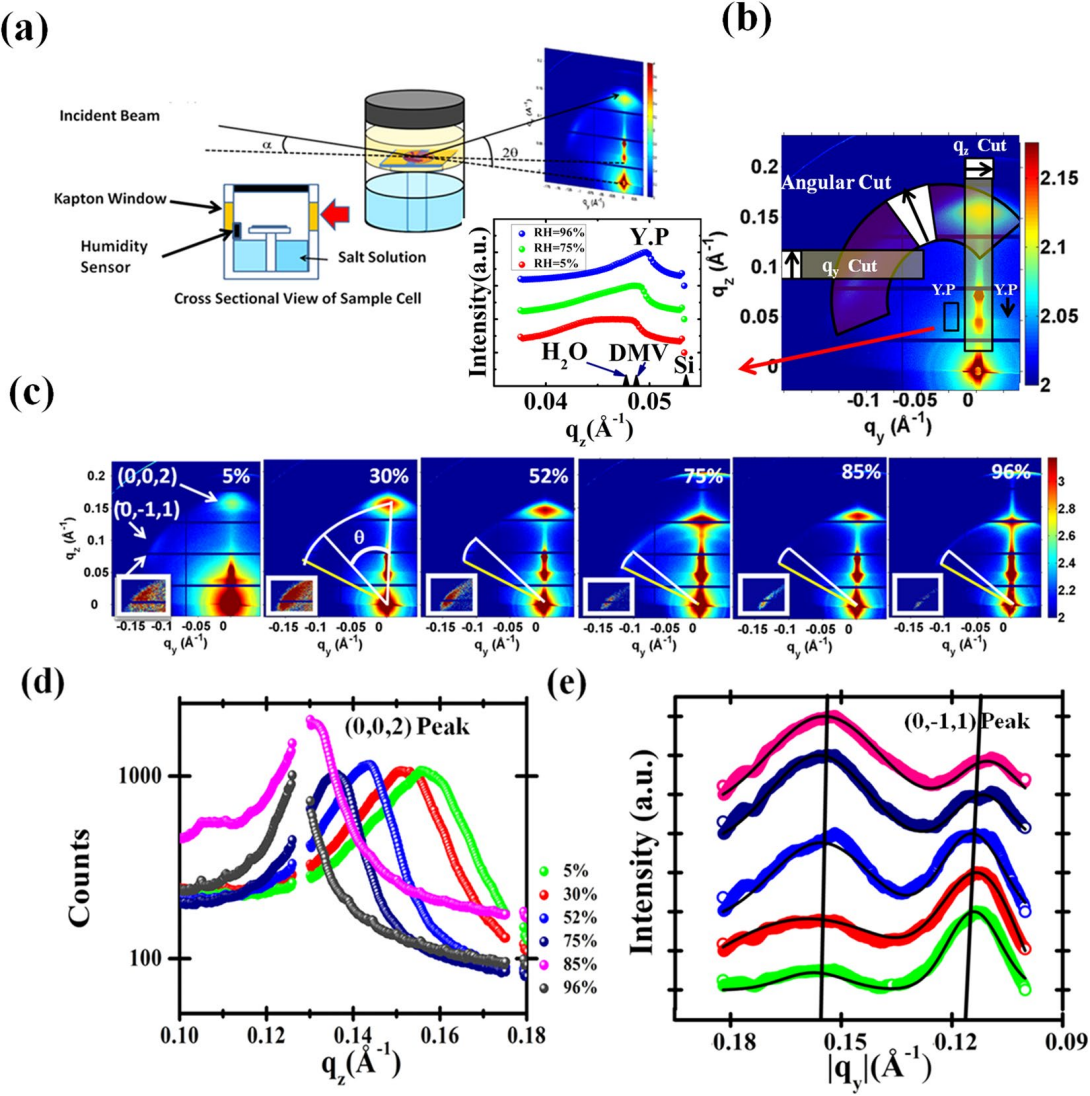
(**a**) Schematic diagram of the sample-cell and experimental arrangement used for RH dependent GISAXS measurements of DMV-CS fibers. (**b**) Different data reduction strategies of the 2D GISAXS data collected in PILATUS 1 M detector into 1D cuts are indicated. The black arrow in the white cell of each box represents the direction of pixel integration. The Yoneda peak (YP) is observed in specular direction and also as a horizontal line along q_y_ direction. A representative linear plot of YP along q_z_ obtained in various humidity conditions are also shown - these plots were generated by integrating along q_y_ direction as indicated by the black outlined rectangle. The expected YP positions of water, DMV and silicon-substrate calculated from the known electron densities are also marked here. (**c**) RH dependent GISAXS data of DMV-CS nanofibers on Si substrate. The two diffraction spots are indexed as (0, 0, 2) and (0, −1, 1) (refer insets for magnified scale) and the angular separation θ of spots as indicated in lattice model shown in Fig. 3(d) are indicated - the position of perfect hexagonal lattice (yellow line) is indicated and the white line gives the position of the observed spot. The gradual reduction in the distortion with increase in RH values given is apparent. (**d**) and (**e**) show RH dependent intensity profiles along the vertical (q_z_) and horizontal (q_y_) direction as obtained from the 2D detector data. The (0, 0, 2) peak is apparent in q_z_ data and q_y_ data reveals presence of two peaks, first peak correspond to a lamellar structure and second peak is the (0, −1, 1) reflection.

The grazing incidence small angle x-ray scattering (GISAXS) technique used for this study is an ideal method to monitor self-organization process of the internal structure of such systems^39–41^. The DMV-CS films formed through drying of drop-casted dispersion on a substrate allowed us to carry out *in-situ* GISAXS measurements in two different ways though the films obtained by drying exhibit much higher unevenness (refer optical images in the section “Self assembly of nanofibers during drying”) as compared to the spin-coated films (refer AFM image in Fig. 1). The large variation in the diameters of the nanofibers resulted in a very rough film of about 2 micron thickness and the measured Yoneda peak (YP in Fig. 2(b)) and diffused scattering was found to be sensitive to the position of the footprint of the x-ray beam. It is apparent in the cut along the q_z_ direction of the YP shown in Fig. 2(b) that in high humid condition the film become smoother and the Yoneda peak becomes well defined. We have also indicated in this figure the expected YP positions of water, DMV and silicon-substrate calculated from the known electron densities. The electron density of DMV-CS was found to be higher than that of reported DMV electron density - more study is required on this subject with the help of smother film and/or sub-micron beam to reduce x-ray footprint. In this work we concentrated on the analysis of Bragg peaks using Born approximation assuming an effective surface^42–50^ as elaborated in the methods section. In the first set of experiments, GISAXS measurements were carried out on already formed films of nanofibers in controlled environment by varying the RH value from 5% to 96%. In the second set, the self-assembling process of nanofibers during drying was monitored as water evaporates leaving behind a film of nanofibers on a substrate - we made sure that the footprint of x-ray beam remains within the covered area of the films. The results obtained from these two sets of experiments provide an insight into the humidity dependent formation of the nanofiber-bundles through the self-assembly process. We plan to carry out in future GISAXS study of individual nanofibers with sub-micron x-ray beams^48^.

## Results

### Aridity dependent distortion of nanofibers

**Figure 3:**
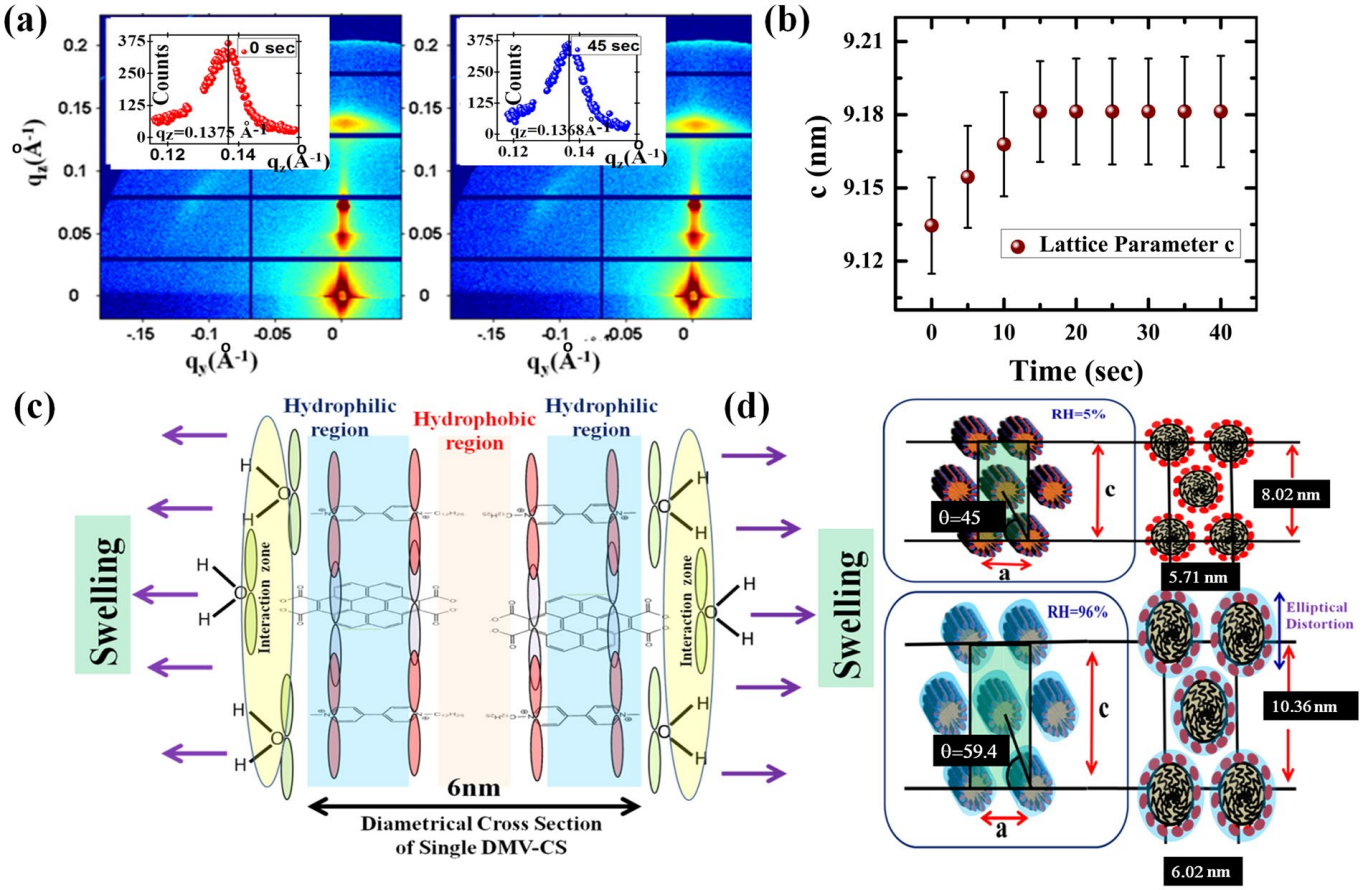
(**a**) GISAXS images of DMV-CS film during transition from RH 52% (left) to RH 75% (right) with insets showing the (0, 0, 2) peak along q_z_ direction. The quick change in the lattice parameter ‘**c**’ with time is shown in the extreme right panel with error bars as the RH value is changed from 52% to 75% - equilibrium state is achieved in about 20 Sec. (**c**) Schematic of the cross section of a nanofiber with two DMV-CS molecules arranged back to back along the diameter of a typical micelle. The hydrophobic zone is in the interior of the micelle with the hydrophobic hydrocarbon chains of DMV molecules facing towards each other. The hydrophilic zone is the upper layer of the cylindrical shell that form DMV-CS charge transfer complex as indicated. (**d**) Internal structure and structural exfoliation of DMV-CS near Hexagonal lattice which was analyzed with a Face Centered lattice with the in plane and out of plane lattice parameters a and c, respectively. The angle between the (0, 0, 2) and (0, −1, 1) plane reflection θ becomes 60° as this lattice become perfect hexagonal lattice with c = √3a.

In Fig. 2(a) and (b), we have presented a schematic diagram of the experimental arrangements. A cylindrical cell having sample on silicon substrate (refer Fig. 2(a)) and Kapton windows for x-ray transmission, was used for both types of measurements. The grazing angle of incidence α was set to 0.42° giving only one specularly reflected spot on the two dimensional (2D) detector (PILATUS 1 M) at scattering angle 2α giving a finite value of q_z_ = (4π/λ)*sin*α and at this spot in-plane components of the wave-vectors, namely q_y_ and q_x_ remained zero. For all other pixels on the detector, all the three components of the wave vector **q** (q_x_, q_y_, q_z_) have finite non-zero values. In this geometry, the q_z_ and q_y_ values change rapidly in vertical and horizontal directions, respectively, as shown in Fig. 2(b). In Fig. 2(b), we have also indicated the data reduction strategies employed for obtaining one dimensional (1D) intensity profiles to carry out quantitative analysis. Few pixels were integrated near q_y_ = 0 to generate such 1D data along the q_z_ direction termed as q_z_ cuts (refer Fig. 2(b)). Similarly the angular cuts, providing the intensity distribution as a function of scattering angle θ, were generated from the measured 2D data. We also generated q_y_ cuts near the peak close to θ = 60°, for the analysis of less intense peaks. The near hexagonal symmetry present in the nanofibers is apparent from the raw 2D data itself shown in the Fig. 2(c) as a function of the RH. The diffraction spots observed along the q_z_ and q_y_ directions could be indexed as (0, 0, 2) and (0, −1, 1) peaks. We could obtain the (0, 1, 1) peak on the right-side as well, by moving the detector appropriately. The q_z_ value of the (0, 0, 2) peak showed systematic reduction as the equilibrium RH was increased from 5% to 96% due to swelling of the hexagonal lattice of DMV-CS nanofibers. We have shown in Fig. 2(d) the 1D intensity profile as a function of q_z_ around this (0, 0, 2) peak as indicated in Fig. 2(b). The width of this peak reduces from 0.19 nm^−1^ to 0.09 nm^−1^ as the RH is changed from 5% to 85%. It is also clear in Fig. 2(c) that the (0, −1, 1) peak occurs at smaller angles (less than θ = 60°) with respect to the (0, 0, 2) peak in arid condition (refer white and yellow lines representing measured and 60° positions respectively in Fig. 2(b)). As the equilibrium RH value increased from 5% to 96%, the measured angle θ approaches 60° giving symmetric hexagonal pattern. The (0, −1, 1) peak has also been shown in higher magnification for better clarity in the insets of Fig. 2(c). The q_y_ cuts of this peak are shown in Fig. 2(e) - the width of this peak did not show any significant change with variation in the RH. Although it is not apparent in the GISAXS data shown in Fig. 2(c), we could extract an additional powder-like peak near the (0, −1, 1) diffraction spot (refer Fig. 2(e)) by integrating the data along q_y_ as shown in Fig. 2(b). As the RH becomes higher, relative intensity of this additional peak with respect to that of (0, −1, 1) peak, increases though the position of this peak remains fixed at the **q** value of 1.54 nm^−1^ (refer Fig. 2(e)). This peak appears due to randomly oriented lamellar phase as we shall discuss in the next section that this lamellar peak first appears at **q** value of 1.4 nm^−1^ and then this peak shift to 1.54 nm^−1^ during formation of the film. The fast response time of the DMV-CS nanofibers with the change in the RH value was monitored by taking GISAXS data every 5 sec. The gradual change in the position of (0, 0, 2) peak during the sampled data revealed the quick response of the material with change in RH value from 52% to 75% (refer Fig. 3(a)). The obtained variation of the lattice parameter c from the analysis of the collected spectra is shown in Fig. 3(b) with error bars. In this dynamic measurement we could see that c-parameter equilibrate within around 20 seconds to the corresponding new RH value even though error bars are high. However the value of c-parameter corresponding to the 52% equilibrium RH could not be obtained in this measurement due to delay in the initiation of data taking.

We have carried out a systematic analysis of the measured data using a simple model of the assembly of DMV-CS nanofibers. In Fig. 3(c) we have shown schematically the cross sectional view of a nanofiber with hydrophobic core made of DMV hydrocarbon tails and the hydrophilic exterior consisting of DMV head-CS charge transfer complex. It is known that the presence of water molecules surrounding the nanofiber surface can alter the π-π overlap in the molecule which is shown schematically in Fig. 3(d) in high RH (lower) and arid (upper) conditions. The Face Centered (FC) lattice shown in Fig. 3(d) is rather a convenient approach^39, 42^ to extract distortions in the hexagonal lattice. The value of the out-of-plane lattice parameter, ‘**c**’, becomes equal to √3**a**, as the base angle defined as *cos* ^−1^(**c**/2**a**), approaches 60° (refer Fig. 3(d)), ‘**a**’ being the in-plane lattice parameter. It is to be noted that (0, 0, 1) peak is extinct in this model. We found that the change in q_z_ value for the (0, 0, 2) peak is much higher (26%) than the change in the position of (0, −1, 1) peak by about 4%. The calculated change in ‘**a**’ and ‘**c**’ parameters with the shift in (0, 0, 2) and (0, −1, 1) peaks give the estimate of the change in the lattice volume per unit length of a nanofiber. For the change in RH value from 5% to 96% the average diameter of the fibers change from 5.7 nm to 5.96 nm resulting in the volume change of a unit cell per unit length of the fiber from 45.7 nm^3^ to 60.4 nm^3^. Considering a single water molecule as a sphere of volume 0.03 nm^3^ and assuming the change in volume is due to introduction of water molecules only, estimated number of extra water molecule come out to be 490 per nanometer length of these fibers. The interaction with these extra water molecules causes the contraction in π-π bond length as observed earlier^37^.

**Figure 4:**
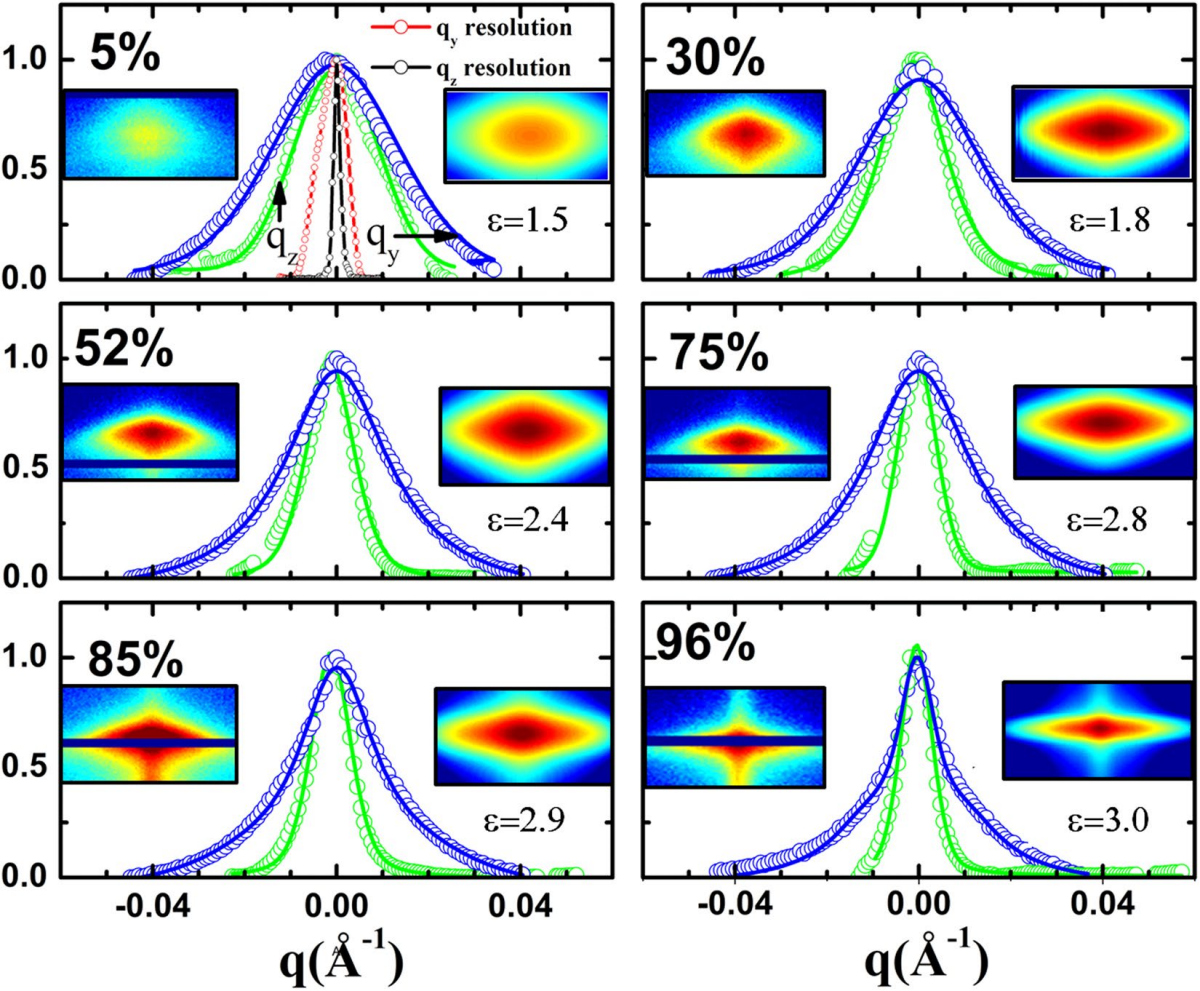
Intensity profile across the (0, 0, 2) spot as a function of humidity along q_z_ (green) and q_y_ (blue) directions plotted together in the same scale. The measured resolution functions in the two directions are shown in top left frame. The peaks were fitted using pseudo Voigt function. The measured and the calculated intensity-shape of the peaks are shown for all the RH values along with the obtained eccentricity determined from peak width-ratio. In each panel we have shown calculated intensity-shape I(q_y_, q_z_) of (0, 0, 2) spot with the measured shape. Calculated shapes represent the measured data well – the associated eccentricity ε for each RH values are also shown in each panel.

The shape evolution of the individual micelle can be intuitively derived from the notion of Curie principle^43^ of scattering which stipulates that the symmetry of the measured diffraction spot must be identical to the symmetry of the scatterer. The form factor (refer equation 2 in methods) of the individual micelle plays a defining role in the shape of the observed diffraction spot. It is evident from the intensity contour of the (0, 0, 2) peak (refer Fig. 2(c)) that with increase in RH value, the shape of the spot changes continuously from circular to an elliptical one with major axis along q_y_ direction. This feature indicates that with increase in RH, cross section of the average fiber changes from circular to elliptical shape with major axis in the out-of-plane direction in real space and the elliptical distortion in the DMV-CS nanofibers increase with the RH value of the environment. We extracted intensity profile of the (0, 0, 2) peak along q_z_ and q_y_ directions to quantify this elliptical distortion and calculated^51, 52^ the intensity-shape I(q_y_, q_z_) of the (0, 0, 2) peak using equation 3 in methods. In Fig. 4 we have shown the resolution functions and extracted intensity profiles along q_z_ and q_y_ directions at various RH values. We have also shown in Fig. 4 the measured intensity-shape as a function of RH values along with the calculated intensity-shapes I(q_y_, q_z_) obtained from equation 3. For these calculations we have used Voigt function to obtain better fitting profiles (refer Fig. 4) along the q_z_ and q_y_ directions. The profiles show significant change in the peak-width along the q_z_ direction and sharpening of the central portion of the peak that exhibit long tail along the q_y_ direction as the RH value increase. This effect cannot be due to powder-arc formation as no such shape-change was observed for the (0, −1, 1) peak (refer Fig. 2(c) and (e)). In top left panel of Fig. 4 we have also shown measured resolution functions along q_z_ and q_y_ directions. In each panel we have also shown the obtained width-ratio (ε) of the profiles along q_y_ and q_z_ directions as a function of the RH. The peak-widths obtained from the fitting and from the base-line crossing at 10% of peak-intensity were found to be consistent. It should be noted here that at 5% RH the width-ratio of 1.5 comes primarily due to higher width of resolution function along q_y_ direction - signifying that intensity contour of (0, 0, 2) peak at 5% RH is almost circular in nature. The width-ratio become close to 3.0 at and above 75% RH and the presented results in Fig. 4 clearly show that average cross-section of nanofibers become elliptical with high (>3) eccentricity. It should be noted here that unlike earlier observation^51^ elliptical symmetry in diffraction spots do not appear due to elliptical shape change, here we obtain better hexagonal symmetry as the nanofibers take elliptical shape in the higher RH values. The obtained circular-to-elliptical shape transition was found to be reversible.

### Self Assembly of nanofibers during drying

**Figure 5:**
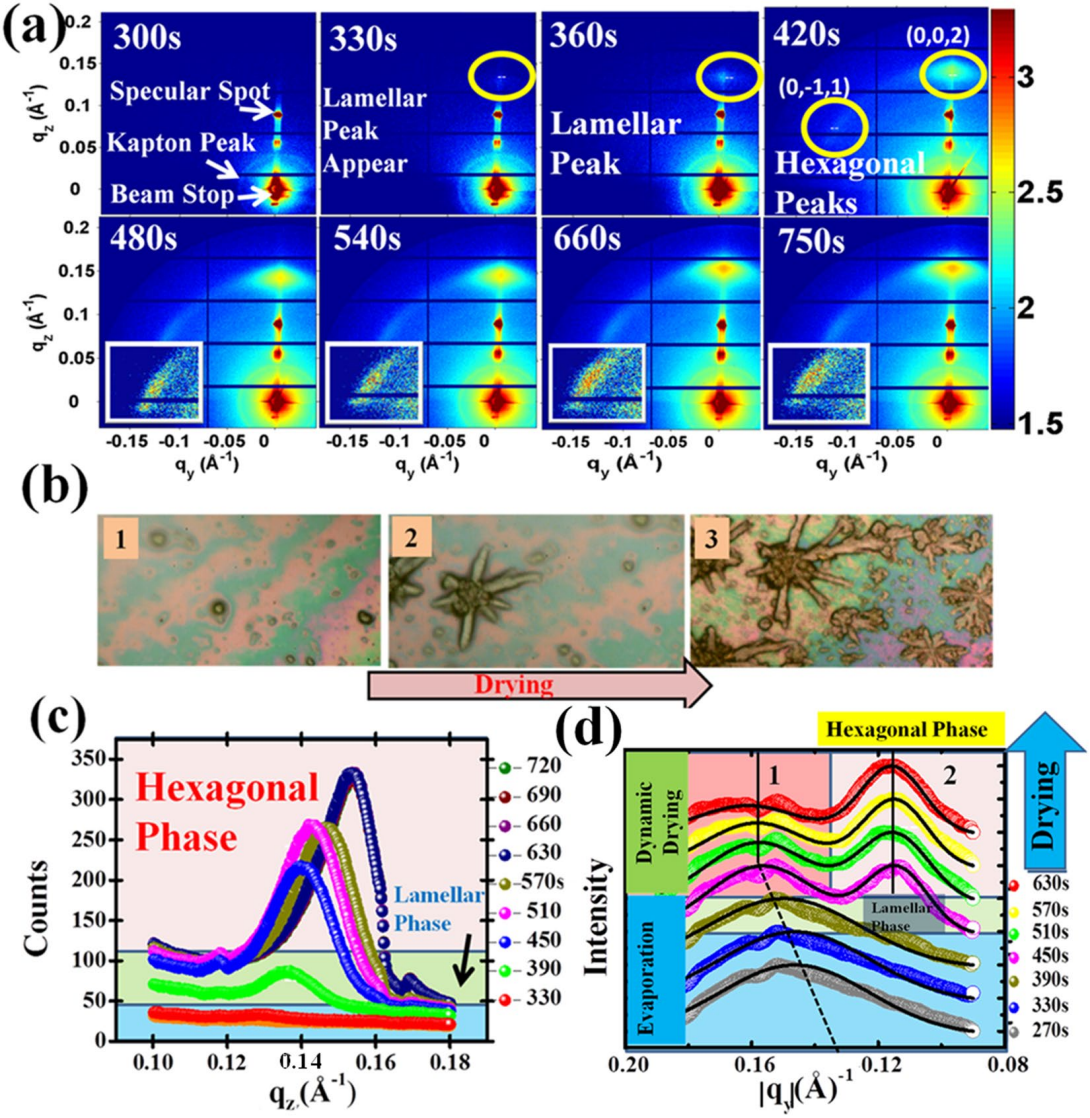
The evolution of DMV-CS nanofibers in the solution as a function of time during drying. (**a**) The representative data reveals three distinct regions, for initial 300 s no ordered structure was observed, then from 330 s to 360 s a single spot in the q_z_ direction was observed indicating formation of a lamellar structure. After 450 s distorted hexagonal structures appear in the drying solution as the (0, 0, 2) and (0, −1, 1) (refer insets for magnified scale) diffraction spots appear. Further evaporation results in the increase in the distortion of the lattice as the water content in the solution decreases. (**b**) The optical microscopy images are shown at three representative stages of the drying process, namely (1) the initial condition when the nanofiber dispersion was deposited on the substrate, (2) the beginning of the formation of structures and (3) the final assembled structure of the nanofibers. (**c**) and (**d**) show the q_z_ and the q_y_ cuts of the 2D GISAXS data respectively where transition from lamellar to hexagonal phase is apparent in both q_z_ and q_y_ cuts. The q_y_ data also elucidate the evolution of the lamellar phase – the shift in the position of this peak is indicated.

*In-Situ* GISAXS measurements were carried out by keeping the x-ray beam on a drop of 2 mM DMV-CS solution over a hydrophobic silicon substrate and the data was collected continuously as the solution dried. Figure 5 depicts the evolution of the internal structure of the DMV-CS nanofibers as a function of drying time. The GISAXS data was taken every 30 seconds in the PILATUS 1 M detector for the *in-situ* monitoring of the drying dynamics and some representative data are shown here. The first frame in Fig. 5(a) shows the initial data taken after 300 seconds of drying and this data do not exhibit signature of any ordered structure, implying that DMV-CS micelles are freely moving with other unassembled or partially assembled conglomerates in the organic matrix. In this frame we can observe only specular spot and powder-ring corresponding to Kapton window. The concentration of the micelle within the drop increases due to evaporation of water and the hydrophilic surface of individual micelles begins to coalesce. A layered lamellar structure become evident in the GISAXS data collected after 330 s as a single isolated spot (refer second frame of Fig. 5(a)) at q_z_ = 1.4 nm^−1^ appear representing the initial ordering of the DMV-CS molecules. The appearance of this spot along q_z_ direction indicates the formation of a lamellar stack parallel to the hydrophilic substrate to minimize the repulsion between the hydrophilic heads and hydrophobic tails. After the drying time of 420 s (fourth frame onwards) with further lowering of solvent concentration the micelles settle into hexagonal lattice structure. The evolving ordering of the DMV-CS molecules during drying process could be followed as the spot positions moves away in q space indicating shrinkage in the lattice parameters (refer q_z_ cuts around (0, 0, 2) peak in Fig. 5(c)). The average diameter of the fibers, obtained from lattice parameter **a**, reduce from 5.77 nm to 5.41 nm as the film becomes completely dry after 750 sec. We could monitor evolution of the initial lamellar peak by generating q_y_ cuts of the GISAXS data taken as a function of drying time. A broad peak (refer Fig. 5(d)) could be detected even in 270 s data at around q_y_ = 1.4 nm^−1^ indicating that all the lamellar stacks are not parallel to the substrate in the DMV-CS dispersion. This peak shifts gradually (refer Fig. 5(d)) towards higher q_y_ and take the value of 1.54 nm^−1^ as also observed in the Fig. 2(e). However in q_z_ cuts (refer Fig. 5(c)) such shift in the position of lamellar peak could not be detected due to the presence of strong (0, 0, 2) peak. The observed change seen in the peak-position implies that with progress in evaporation process the lamellar phase become more compact and the lamellar stacking change from 4.43 nm to 4.1 nm as evaporation time changes from 270 to 390 seconds. Figure 5(d) also show the evolution of (0, −1, 1) hexagonal peak after 400 s of drying time. It should be noted here that the existence of lamellar phase is being proposed based on a single peak obtained in both types of experiments. Detailed structural studies will be required in future to understand this phase.

## Discussion and Conclusions

In optical micrographs shown in Fig. 5(b) one can see evolution of nucleation center and then as the concentration of DMV and CS molecules increases due to water evaporation, the formation of long tubes in star-like dendrimeric structure becomes evident. The GISAXS data obtained during drying experiment also show that initially lamellar structure with periodicity of about 4 nm form and then long fibers showing a hexagonal cross section appear in GISAXS data. Although lamellar peak appears dominantly along the q_z_ direction, this peak could be traced in q_y_ scans as well (refer Fig. 5(d)) indicating that preferred orientation of lamellar phase is parallel to the hydrophilic substrate. As the DMV-CS concentration increase in the solution due to drying, expectedly the peak shifts towards higher q value making the lamellar structure more compact and the q value become 1.54 nm^−1^. The lamellar peak was also observed in the humidity controlled experiment (refer Fig. 2(e)) at this q value.

**Figure 6:**
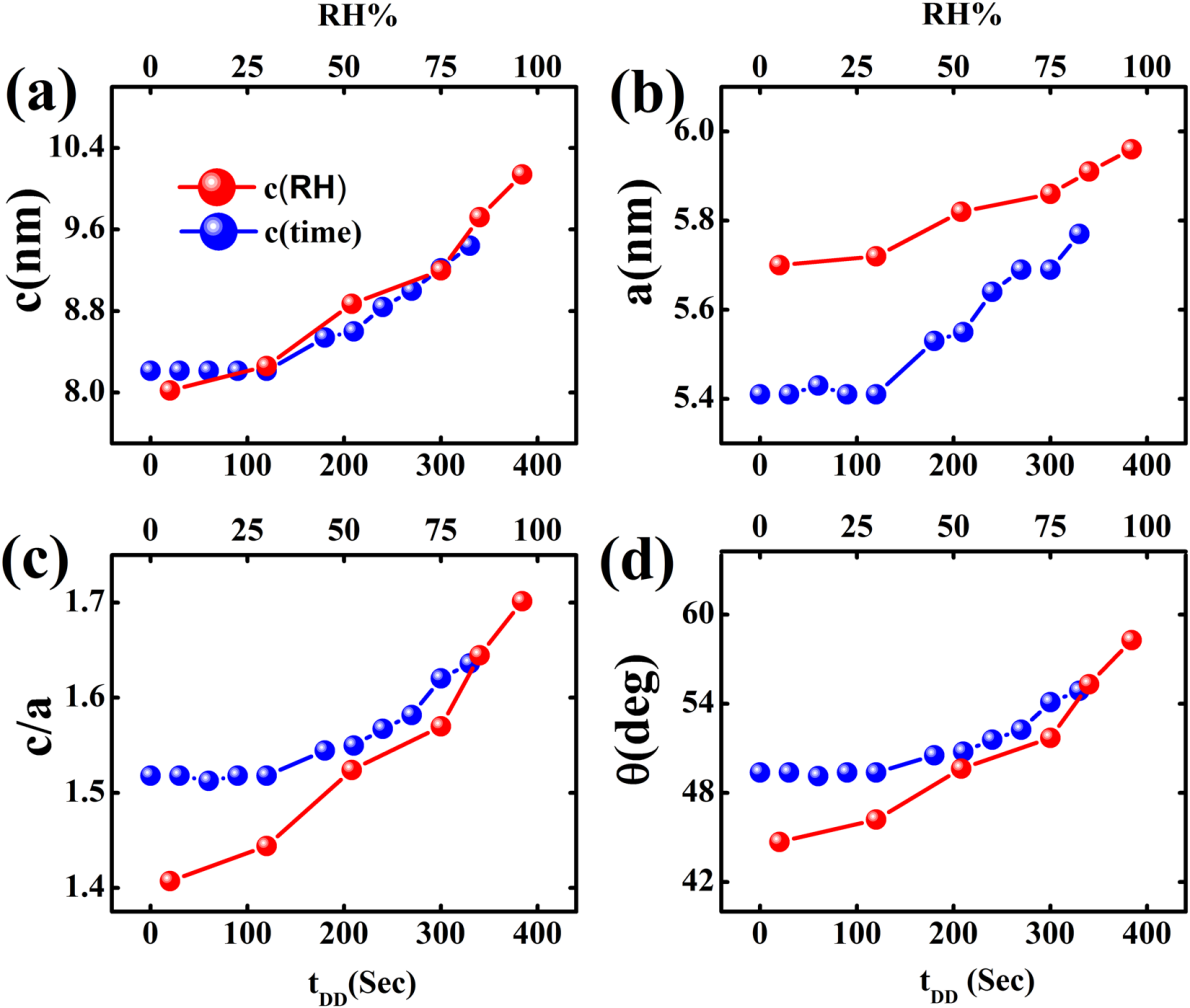
Comparison of results obtained from humidity and drying experiments - for generating the plots together t_DD_ was defined as (750-drying time) in seconds. (**a**) The lattice parameters **‘c’**, (**b**) the lattice parameters ‘**a**’, (**c**) distortion parameter ‘**c/a**’ and (**d**) the angle between the lattice vectors obtained from both types of experiments are plotted together.

We now compare hexagonal ordering of nanofibers as a function of RH and drying time. To facilitate this comparison, we have plotted in Fig. 6 the basic parameters of the distorted hexagonal lattice as shown in Fig. 3(d) showing along x-axis both equilibrium RH values and t_DD_ (=750 – drying time in seconds) with zero representing the end of the drying, as the film is assumed to be completely dry after 750 sec. It should be noted here that ambient RH in the experimental hutch was ~30%, as a result parameters obtained from drying experiment remain unchanged after attaining values corresponding to this ambient RH value. The systematic decrease in ‘**c**’, ‘**a**’, ‘**c/a**’ values of the lattice as the aridity increases is apparent from Fig. 6(a–c) respectively. It is also clear from Fig. 6(c) and (d) that the hexagonal lattice undergoes maximum distortion in lower RH condition with ‘**c/a**’ and angle ‘θ’ tending towards 1.40 and 45° respectively. In high RH condition the distortion in hexagonal lattice almost disappears giving ‘**c/a**’ and θ values as 1.7 and 59.4° respectively. These values are close to the theoretical value of √3 and 60° for an ideal hexagonal lattice structure (refer Fig. 2(c)). We have also found (refer Fig. 4) that elliptical distortion occurs in the shape of the nanofibers as the equilibrium RH increases. Probably existence of water molecules giving rise to swelling distorts the nanofibers in an asymmetric way to tend towards lamellar phase. Although we have observed the existence of randomly oriented lamellar phase in the high humidity data, most of the nanofibers retain hexagonal ordering with highly strained elliptical cross section. This strained condition seems to be the reason of quick response of this material towards lowering of RH conditions. Obviously the sensitivity of this material for humidity will be higher in higher humidity values.

In conclusion, we have presented results of *in-situ* GISAXS measurements to understand the self-organization mechanism of DMV-CS nanofibers as a function of aridity. It was shown that with increase in the humidity, the hexagonal lattice of DMV-CS micelle expands and becomes less distorted. The humidity dependent experiment is conducted on dried film where the anchoring of the nanofibers to the substrate has already taken place. The structural deformation of the distorted hexagonal lattice and elliptical distortion in the individual nanofiber was found to be very sensitive to the environmental RH value. We have carried out model calculations to quantify the observed elliptical shape distortion of the nanofibers anchored to the substrate. It was shown from the drying experiment that the self assembly process exhibit distinct stages of structural evolution. In the first stage as the concentration of DMV-CS within the droplet increase, GISAXS data clearly show the presence of the lamellar phase. Then further drying makes the lamellar stacking evolve to cylindrical nanofibers having a distorted hexagonal structure. In the drying experiment an aqueous solution is drop-casted on a substrate and data is taken during drying of the solution. The elliptical deformation seen in the humidity depended experiment is not observed in the drying experiment as the nanofibers do not get anchored to the substrate before the evaporation of excess water leading to a dry film.

## Methods

### Synthesis of DMV-CS nanofibers

The detailed procedure of synthesis of CS (donor) and DMV (acceptor) molecules has been previously reported^35^. The charge transfer (CT) nanofibers were prepared by adding equimolar aqueous solutions of DMV and CS, by dissolving the salts in Millipore water followed by sonication for 5 minutes and then mixing equal volume of the resultant DMV and CS solutions. The potassium salt of coronene tetracarboxylate (CS) and the dodecyl substituted unsymmetrical viologen derivative (DMV) are used as D and A pairs which interact via ground-state CT interactions to form a hierarchical self-assembly. The concentration of the solvent used in this set of experiments was 2 mM which was optimized by taking RH dependent switching data from the film made with solutions of different concentrations.

### Grazing Incidence Small Angle X-Ray Scattering

Grazing incidence x-ray scattering techniques (GIXS) are extremely useful to determine structure and morphology of top surface and buried interfaces of a thin film^42–54^. X-ray Reflectivity (XRR), Diffuse Scattering and Grazing Incidence Diffraction (GID) are three GIXS techniques that require smooth surface and interfaces of a thin film under investigation. The XRR data measured in specular condition with equal angle of incidence and detection provide information regarding interfacial roughness and electron density profile as a function of depth averaged over in-plane direction. On the other hand diffuse scattering and GID measurements provide us information regarding in-plane morphology and in-plane structure, respectively. It is difficult to obtain smooth films of supramolecular nanofibers on a substrate for obtaining good quality XRR, diffused scattering and GID data. But Grazing Incidence Small Angle X-Ray Scattering (GISAXS) can provide information in such cases and is an ideal nondestructive technique to understand the self-assembly process involved in formation and modification of these supra-molecular nanofibers. It should be mentioned here that with smooth films GISAXS data can provide much more information through measurement of diffused scattering. As the supra-molecular nanofibers formed uneven films, here we concentrated on the analysis of Bragg peaks using Born approximation^42–54^ assuming an effective surface. Moreover as the diameter of the individual micelle in these fibers is around 6 nm one can get diffraction peaks in GISAXS data. The measured x-ray intensity data $I({\boldsymbol{q}})={|A({\boldsymbol{q}})|}^{2}$ on a detector with grazing angle of incidence can be written in Born Approximation as^53–55^

1 $$A({\boldsymbol{q}})={A}_{spec}({\boldsymbol{q}})+{A}_{off-spec}({\boldsymbol{q}})$$

The first term on the right hand side of equation 1 represents specularly reflected beam and valid for only one point on the two dimensional detector where outgoing angle is equal to the incident angle. The second term representing off-specular x-ray scattering data depends on the contrast of the object being studied with the average electron density of the film receiving x-ray beam in the grazing incidence. The GISAXS data can then be expressed as

2 $$A(q)\approx ({\rho }_{object}-{\rho }_{matrix})\ast P({\boldsymbol{q}})\ast F({\boldsymbol{q}})\ast L({\boldsymbol{q}}),$$

where *ρ*_*object*_ is the electron density of the object that is being repeated over real space and *ρ*_*matrix*_ is the average electron density of the film. For the supramolecular nanofibers studied here *ρ*_*object*_ represents the electron density of the radial cross section of a nanofiber and we obtain sharp diffraction peaks in the x-ray detector for ordered fibers. In the above equation 2, P(**q**), F(**q**) and L(**q**) represents the form factor of the individual lattice elements, the structure factor of the lattice and the resolution function. It should be noted here that unlike in conventional diffraction experiment the form factor *P*(***q***) decides here the shape of the measured diffraction spot as the dimension of the scattering object is few nanometer wide. Moreover asymmetry in the scattering object can get reflected in the measured shape of the diffraction spots, as shown earlier^43^. In this geometry $L$(**q**) can be considered as resolution function and the distribution of the diffraction spots on the x-ray detector is essentially determined by the structure factor *F*(***q***) that represents the symmetry of the unit cell. For example in the present experiment we obtain diffraction spots having hexagonal symmetry once the self assembled nanofibers get organized in a hexagonal lattice in radial direction. The observed intensity-shape of the (0, 0, 2) peak on the detector were approximated^51^ by a two-dimensional Gaussian function as

3 $$I({q}_{y},{q}_{z})={I}_{0}exp\{-\frac{1}{2}[\frac{{({q}_{y}-{q}_{y}^{^{\prime} })}^{2}}{{\sigma }_{y}^{2}}+\frac{{({q}_{z}-{q}_{z}^{^{\prime} })}^{2}}{{\sigma }_{z}^{2}}]\}+B.$$

I_0_ is the maximum intensity and B is the constant background; σ_y_ and σ_z_ represent the width of the peak along horizontal (q_y_) and vertical (q_z_) directions – we have neglected any misalignment of the peaks along this coordinate system. The position of the peak in horizontal and vertical direction is given by ${q}_{y}^{^{\prime} }$ and ${q}_{z}^{^{\prime} }$ respectively. We have neglected^49, 51^ the curvature of the Ewald sphere here in the small angle approximation. The intensity-shape I(q_y_, q_z_) as a function of RH values of the (0, 0, 2) peak were calculated using equation (3) after extracting the profile of the peak along q_y_ and q_z_ directions. For better representation of data we used Voigt function instead of the Gaussian function mentioned above – the results of the calculations are shown in Fig. 4.

The GISAXS measurements reported here were performed in the Indian Beamline at Photon Factory, KEK Japan. The sample was kept on a silicon substrate and was illuminated using a monochromatic x-ray of energy 10.138 keV with a sample to detector distance of 3902 mm (refer Fig. 2). The beam dimension was 0.3 mm and 1mm in the vertical and horizontal direction respectively and a fixed incident angle of 0.42° was used. A PILATUS-1M detector with a pixel resolution of 172 µm × 172 µm was used for this study.

The drying experiment was performed with about 10 µL drop of 2 mM solution spread over the pre-aligned substrate covering ~1 cm^2^ area and the foot-print of the beam was kept much below than the formed-film of the cylindrical micelles. A temporal evolution with time snapshots of 30 s was continuously monitored for 20 min - time normally taken by the aqueous dispersion to dry with the atmospheric RH of 30%. We used a sample cell made with aluminum having kapton windows for transmission of incident x-ray beam and scattered beam from the sample kept at the middle of the cell. The internal free volume of the container was used for storing different saturated salt solutions having different equilibrium vapor pressures. Arduino Uno interfaced RH sensor DTH11 was used to monitor and calibrate the cell. For 5% RH a closed dry cell with a flow of dry Helium gas was used. The ambient RH value inside the hutch was 30% and we obtained 52%, 75%, 85% and 96% RH using saturated salt solutions of Mg(NO_3_)_2_, NaCl, KCl and K_2_SO_4_ respectively. All the chemicals were purchased from WAKO Chemicals Japan and were of high purity. The equilibrating time after applying the solution in the cell to alter the RH was found to be approximately 15 min - GISAXS data was collected after this waiting time and RH value remained within 1%.
